# Supplementary material for: Transcriptome and Metabolome Profiling Provide New Insights into Disuse Muscle Atrophy in Chicken: The Potential Role of Fast-Twitch Muscle Fibers
Source: Int J Mol Sci. 2024 Mar 20;25(6):3516. doi: 10.3390/ijms25063516 (PMC10971063; doi:10.3390/ijms25063516)
Supplement: Supplementary file 1 [file ijms-25-03516-s001.zip › Table S1.pdf]

Table S1. Primers used for qPCR

Fragment name	Fragment sequences(5' to 3')	Size (bp)
Atrogin-1(F)	TCAACGGGTCGGCAAGTCT	201
Atrogin-1(R)	TCCCTCCCATCGCTCAGTC	
MURF1(F)	GGACGAGCGGATCAACAT	218
MURF1(R)	GGGAGATGATGGTCTGGATG	
SOX6(F)	TCAGGTTCAGGGTCACATGCC	179
SOX6(R)	TTGCTGGAGCTGTAAAGGGC	
TNNC1(F)	GTTGAGCAGTTGACAGAAGA	180
TNNC1(R)	GAACCATCATAACAAGGAAC	
TNNC2(F)	GAGCAGCAAAGATGGCGTCA	222
TNNC2(R)	ATCACCGTGCCCAACTCCTT	
TNNI1(F)	GAGGAGTGGGAGCAGGAGAT	194
TNNI1(R)	TTCGTCCACAATCTCAACCT	
TNNT1(F)	GAGCCGCACGGAGAAGGAGC	197
TNNT1(R)	CCCGAAGTGGGGCATGTTGG	
PGAM1(F)	GCGAGGCTCAGGTGAAGAT	150
PGAM1(R)	GTCCTCCGTCAGGTCAGC	
PGK1(F)	CCCTGGATAAGGTGGATG	186
PGK1(R)	TTGTCAGGCATGGGAACT	
PYGL(F)	ACATTTGCCTACACGAACC	204
PYGL(R)	TGCCTCCCTCCTCTATCA	
GPI(F)	ATTCACTTTGGGAGCAATC	217
GPI(R)	ACTCCAACTCTGGCTCAAT	
HK1(F)	CTGGATCTCGGTGGTTCTTAC	168
HK1(R)	TTGTCGGCACGGGAAAGA	
CPT1(F)	GCTTATTGTAGTTGTGGGTG	175
CPT1(R)	AAAGTTTGCCGTGTTCAG	
FASN(F)	CGCAGGCATAGCAGGAAA	195
FASN(R)	CCAAAGAAGGAGGCATCAA	
OXSM(F)	ACATAACAGCACCTAATCC	183
OXSM(R)	ATGGGACGTAATTGAGAT	
COX2(F)	GTAGATGCCCAAGAAGTT	192
COX2(R)	GTTTGATTTAGTCGTCCAG	
β-globin(F)	CAGCCAGGTGGAGGATTT	188
β-globin(R)	GAATAGGAGGACCCTCTGTTAG	
β-actin(F)	GATATTGCTGCGCTCGTTG	197
β-actin(R)	TTCAGGGTCAGGATACCTCTTT	
