# Supplementary figures and images for: Transcriptome and Metabolome Profiling Provide New Insights into Disuse Muscle Atrophy in Chicken: The Potential Role of Fast-Twitch Muscle Fibers
Source: Int J Mol Sci. 2024 Mar 20;25(6):3516. doi: 10.3390/ijms25063516 (PMC10971063; doi:10.3390/ijms25063516)

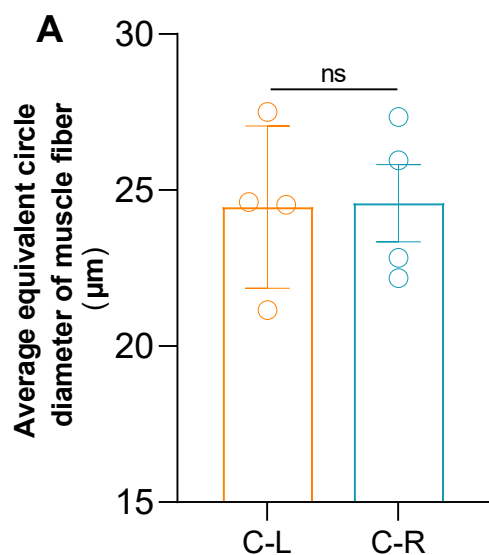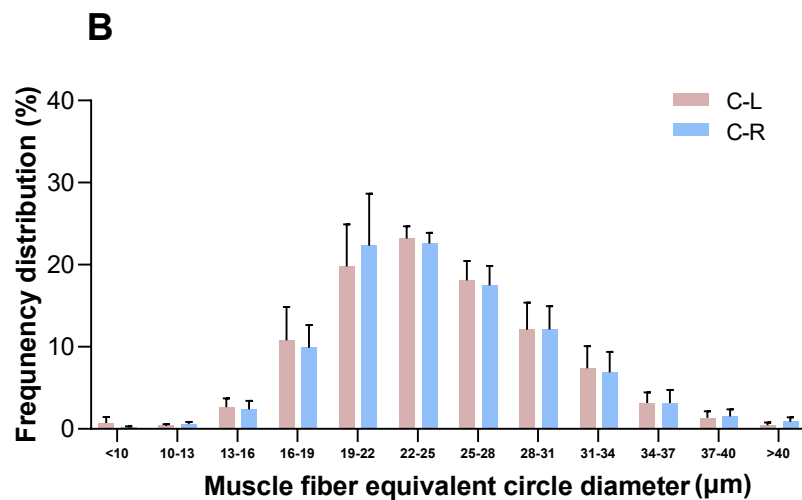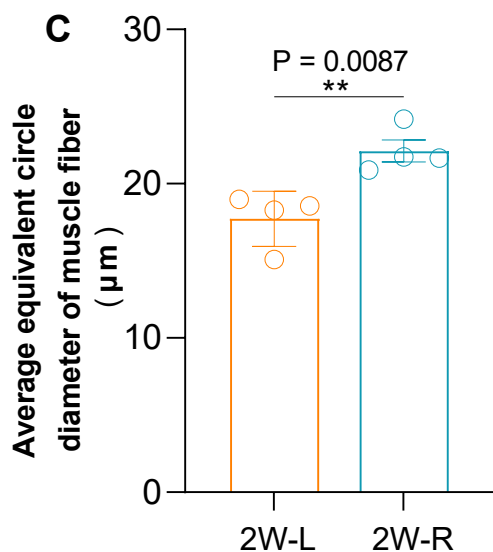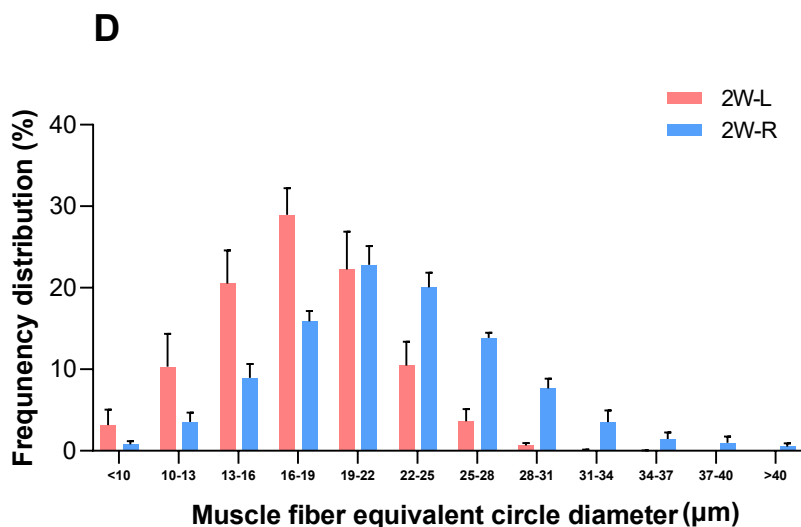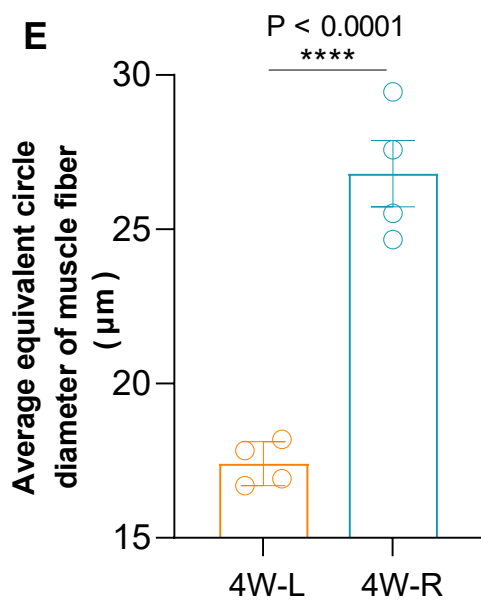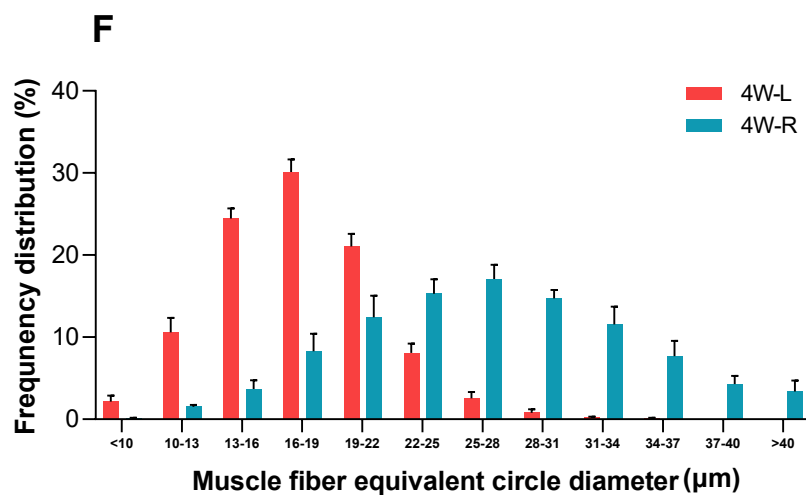

Supplement: Supplementary file 1 [file ijms-25-03516-s001.zip › Figure S1.pdf]

**A**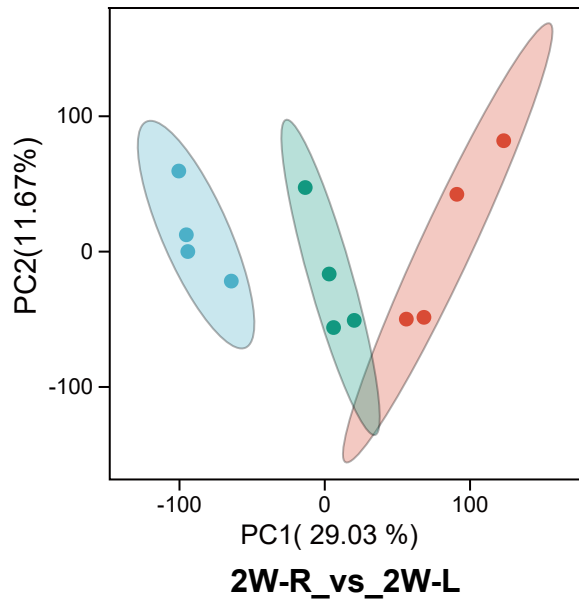**B**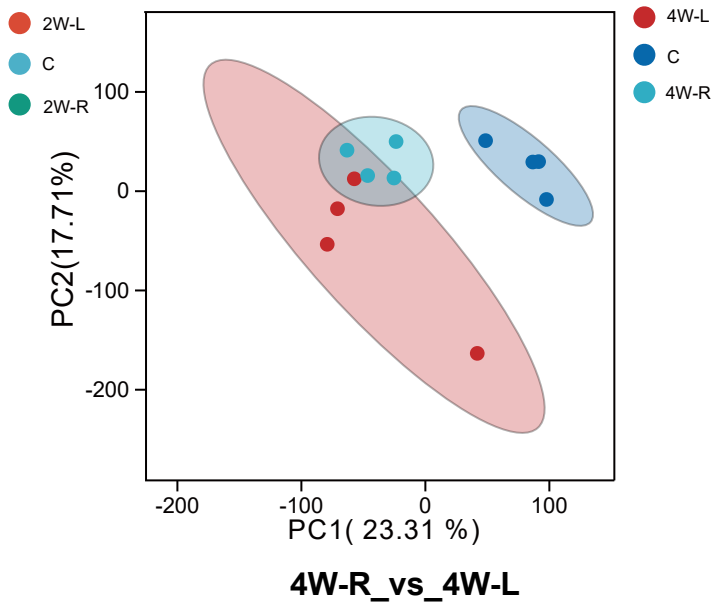

Supplement: Supplementary file 1 [file ijms-25-03516-s001.zip › Figure S2.pdf]

A

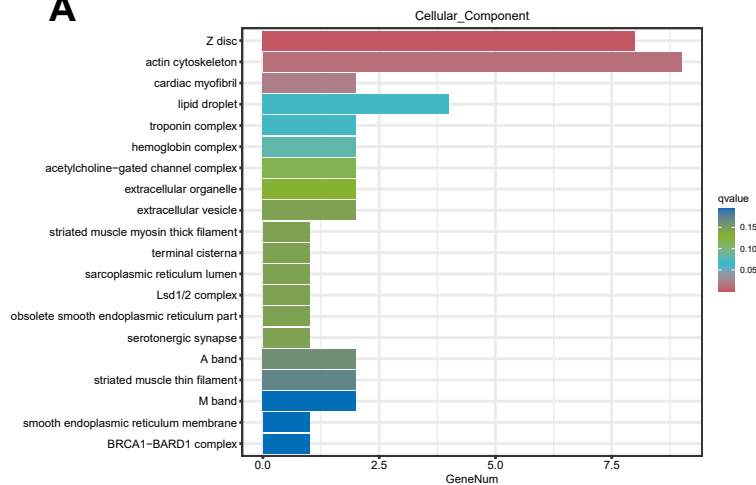

2W-R\_vs\_2W-L

B

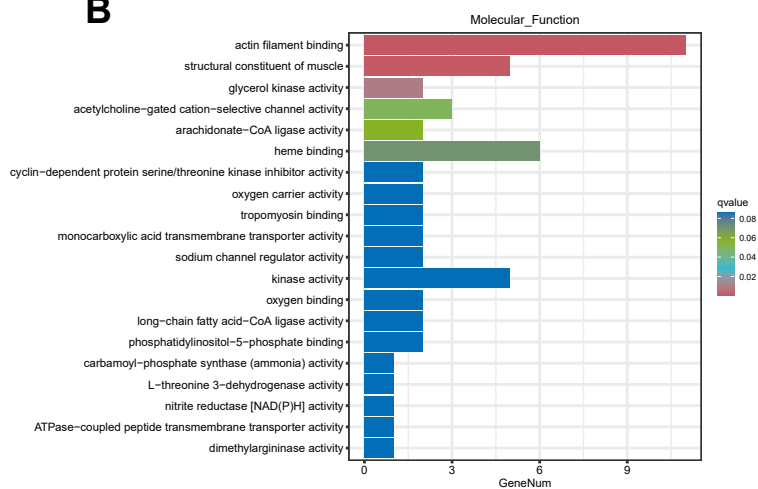

2W-R\_vs\_2W-L

C

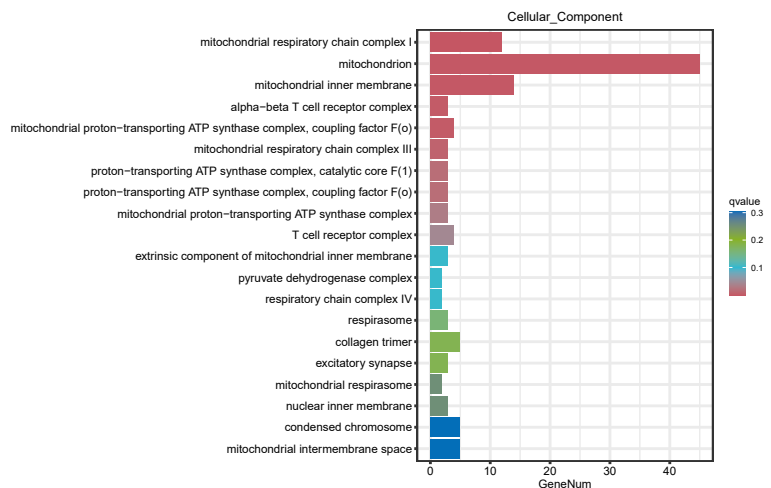

4W-R\_vs\_4W-L

D

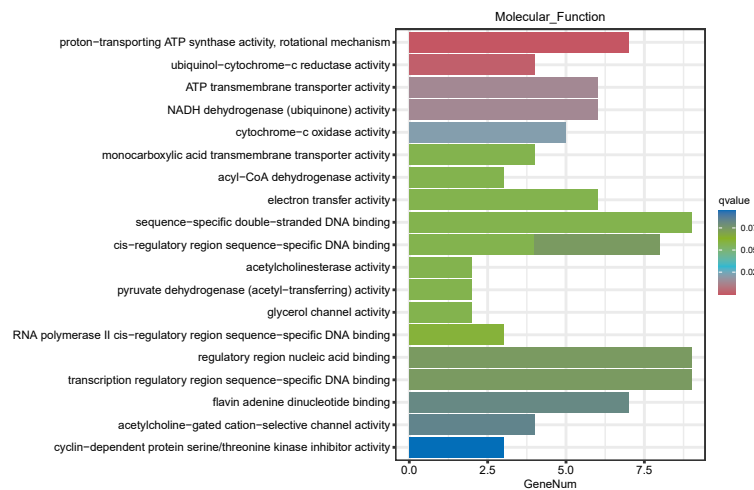

4W-R\_vs\_4W-L

Supplement: Supplementary file 1 [file ijms-25-03516-s001.zip › Figure S3.pdf]
